# Septic Arthritis Caused by Perirectal Abscess

**DOI:** 10.7759/cureus.13343

**Published:** 2021-02-15

**Authors:** Mihir Odak, Steven Douedi, Arsam Nadeem, Anton Mararenko, Swapnil V Patel

**Affiliations:** 1 Internal Medicine, Jersey Shore University Medical Center, Neptune City, USA

**Keywords:** septic arthritis, perirectal abscess

## Abstract

Septic arthritis is a debilitating, inflammatory condition of joints that can cause patients to experience significant pain and discomfort. An estimated 0.006% of people annually develop the condition; the absence of prompt diagnosis and treatment can lead to long-term joint dysfunction. Septic arthritis is usually caused by hematologic seeding of the joint space by bacteria, however, those who receive intra-joint injections and IV access are also at high risk for developing the condition. In this article, we present the case of a 58-year-old male with no immediately identifiable risk factors for septic arthritis, who was found to have a septic joint as a result of an invading perirectal abscess that was discovered in an imaging study.

## Introduction

Septic arthritis is an inflammatory syndrome that leads to severe pain and damage to joints with associated significant morbidity and mortality [1-2]. It is commonly a sequela of bacterial infections that result in colonization of the joint space by the hematologic spread of bacteria to the joint [3]. Annually, approximately up to six out of every 100,000 people are afflicted by septic arthritis, making it a relatively uncommon occurrence [1]. Populations that are at high risk are the elderly, those with compromised immunity, such as poorly controlled diabetes mellitus and human immunodeficiency virus (HIV) infection, as well as patients with recent joint surgeries, skin infections, or IV drug use history [1].

In addition to hematologic spread, there are a number of sources in the pelvic region that can predispose patients to develop septic arthritis of the hip joints. These include the rectum and genitourinary tract. Septic arthritis secondary to extension of perirectal abscesses has been underreported in literature. Here we present a case of septic arthritis of the hip joint in an individual whose only complaint was groin pain, who was subsequently found to have multiple perirectal and pelvic abscesses with extension to the femoral joint space. Our hope in presenting this case is to raise the index of suspicion for abscess etiology in suspected septic arthritis cases, as many of these cases involve therapy beyond the joint itself.

## Case presentation

The patient is a 58-year-old male with a past medical history of insulin-dependent type 2 diabetes mellitus, who presented to our emergency room with a complaint of groin pain. He reported this pain which began one month prior to presentation and has been progressively getting worse. The pain was localized to the left groin with radiation down the left leg. The patient initially thought his pain was due to sciatica because of which he did not seek medical treatment. He endorsed pain while at rest and particularly with movement of the left leg and walking. He denied any complaints of fevers, chills, chest pain, shortness of breath, abdominal pain, nausea, vomiting, diarrhea, flank pain, dysuria, or hematuria. He also denied any incontinence of urine or bladder.

Upon presentation to the ED, the patient’s vital signs were a blood pressure of 134/81 mmHg, heart rate of 75 beats/min, 98.2°F, and oxygen saturation of 99%. His physical examination was significant for sacral pain on palpation, back pain, and abnormal gait. His laboratory studies are shown in Table 1.

**Table 1:** Laboratory values. BUN, blood urea nitrogen; WBC, white blood cell

Laboratory test	Value	Reference
WBC	14.1 x10^3^/uL	4.5–11.0 x10^3^/uL
Hemoglobin	11.5 g/dL	13.2–17.5 g/dL
Platelets	602 x10^3^/uL	140–450 x10^3^/uL
Sodium	130 mmol/L	136–145 mmol/L
Potassium	4.2 mmol/L	3.5–5.2 mmol/L
Chloride	97 mmol/L	96–110 mmol/L
Bicarbonate	24 mmol/L	24–31 mmol/L
Blood urea nitrogen	10 mg/dL	5–25 mg/dL
Creatinine	0.83 mg/dL	0.61–1.24 mg/dL
Glucose	337 mg/dL	70–99 mg/dL
Urinalysis	Urine glucose: >500 Urine bilirubin: negative Urine ketones: negative Specific gravity: 1.023 Urine blood: small Urine protein: negative Urine nitrites: positive Urine leukocytes: large	Urine glucose: negative Urine bilirubin: negative Urine ketones: negative Specific gravity: 1.005 – 1.030 Urine blood: negative Urine protein: negative Urine nitrites: negative Urine leukocytes: negative

**Figure 1:**
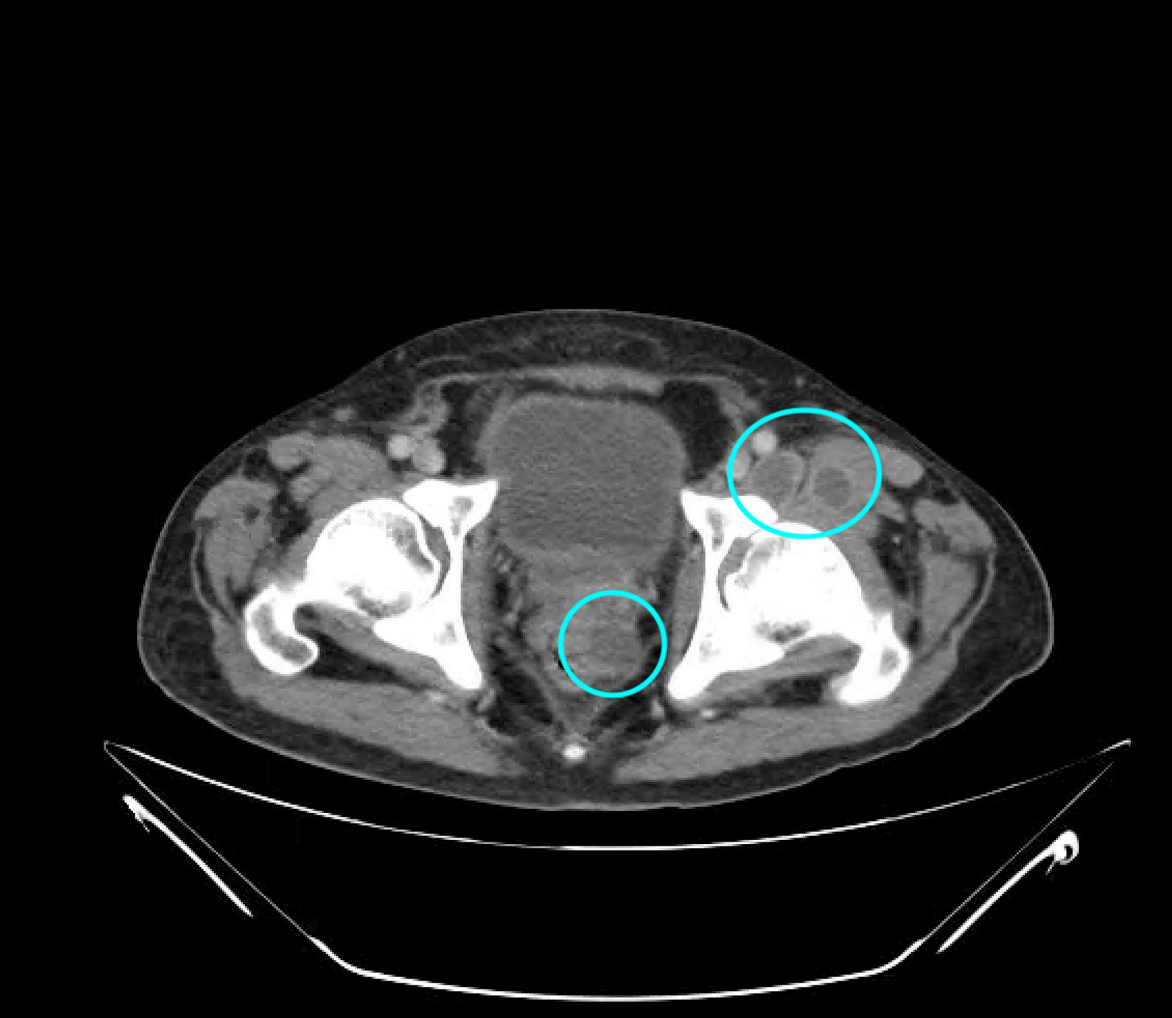
CT abdomen/pelvis showing fluid collections in the left anterior hip and perirectal area (blue circles).

**Figure 2:**
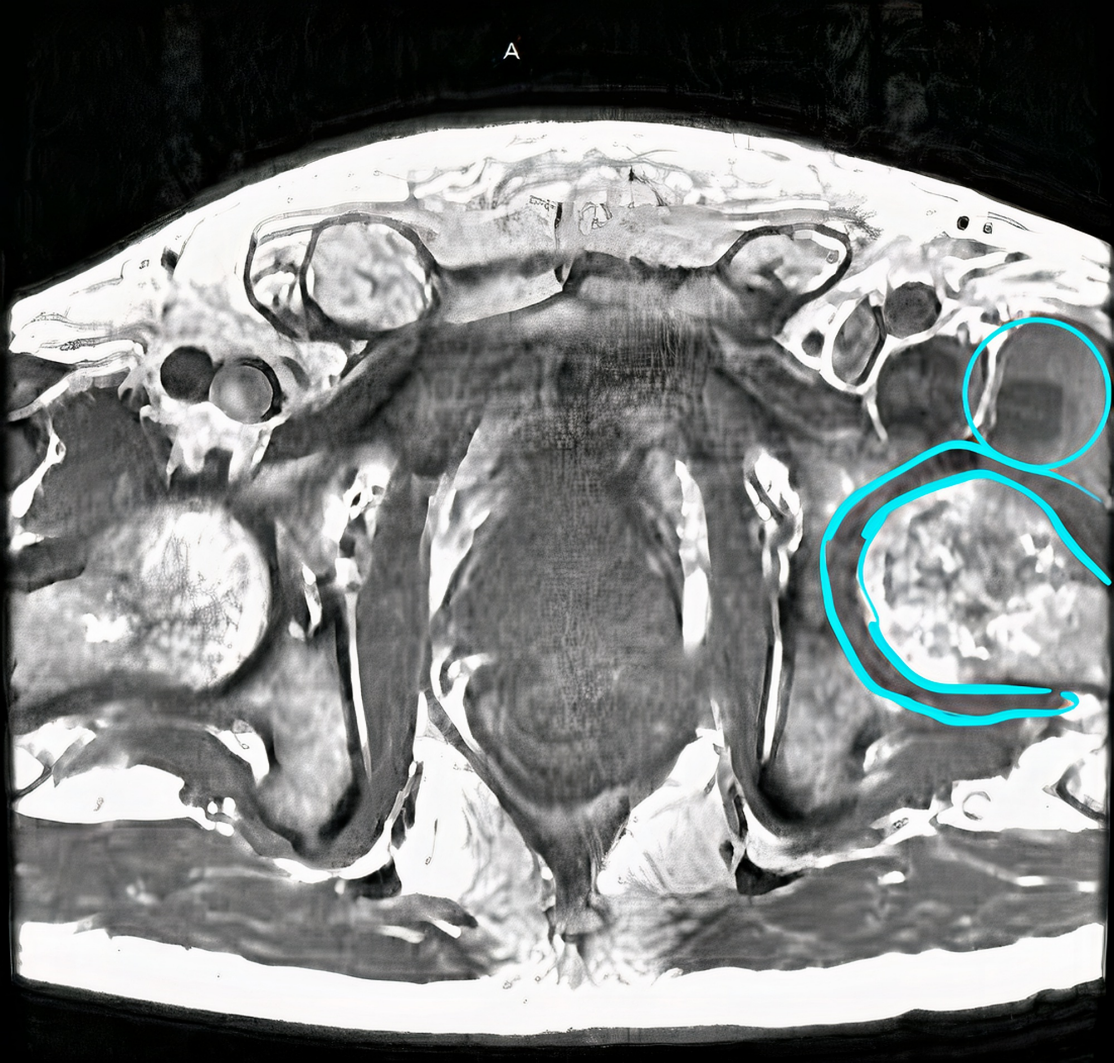
MRI abdomen/pelvis showing left anterior abscesses (blue circle) and joint capsule inflammation (blue outline).

**Figure 3:**
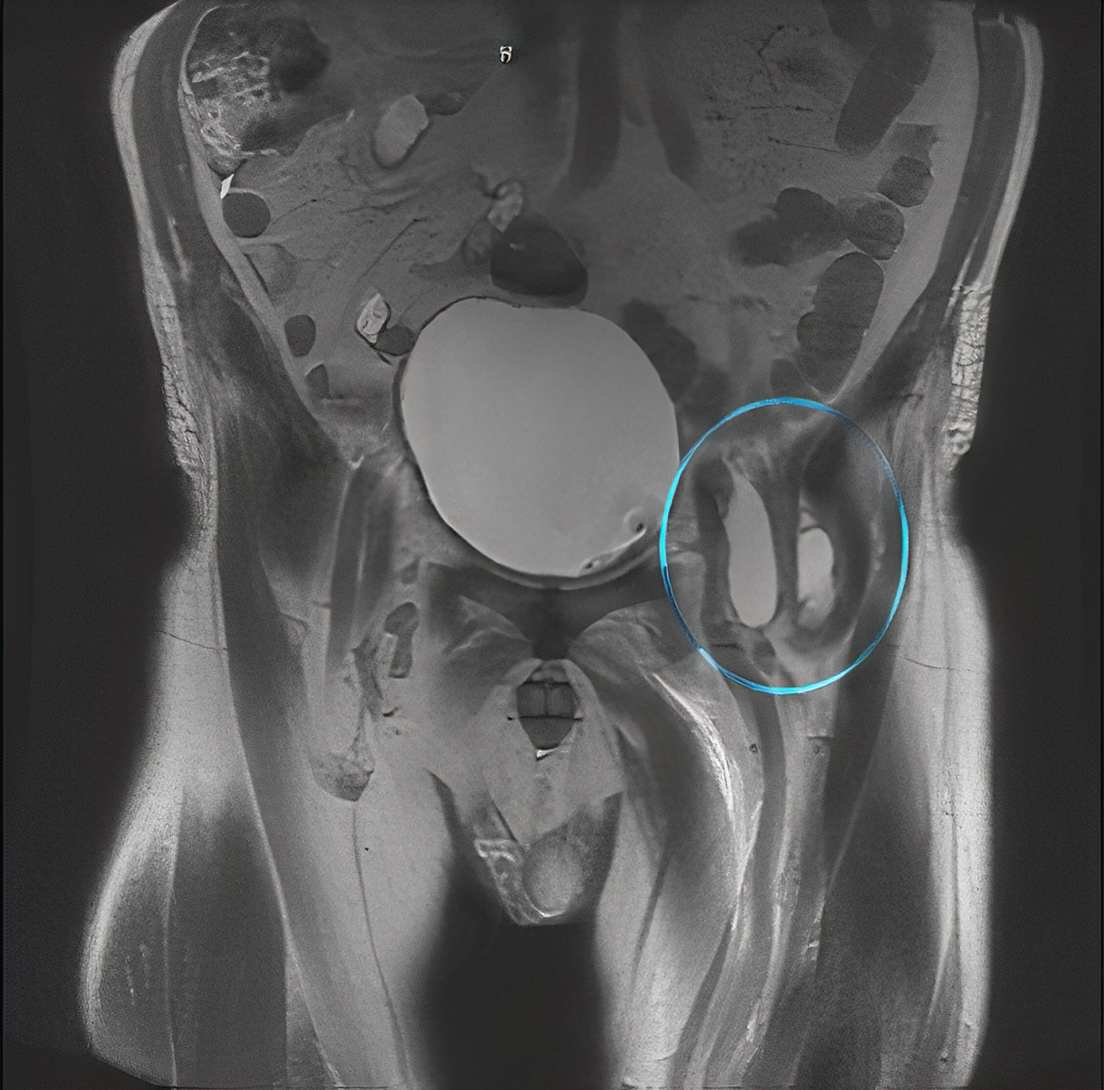
MRI abdomen/pelvis showing abscess extension to the left anterior hip (blue circle).

**Figure 4:**
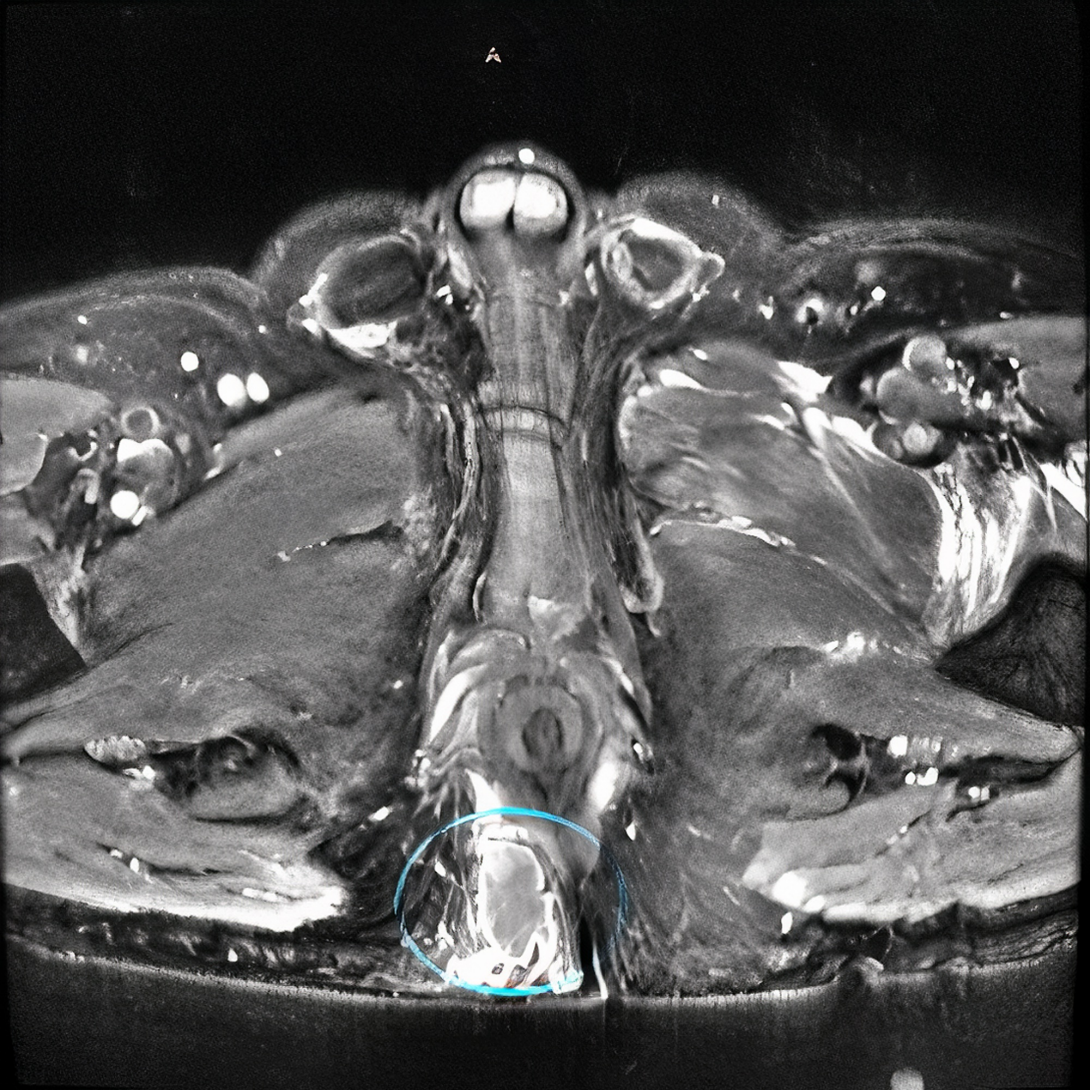
MRI abdomen/pelvis showing perirectal abscess (blue circle).

He was administered vancomycin and piperacillin-tazobactam and taken for a CT scan (Figure 1) which revealed a possible prostatic abscess with extension to the ischiorectal fossa and right gluteal region. Two fluid collections were also identified in the anterior aspect of the left hip. An MRI was also performed (Figures 2-4), which revealed multifocal perirectal abscesses with extension to the inferior ischiorectal fossae, right greater than left. Abscesses were also seen at the anterior aspect of the left hip indicative of septic arthritis. The patient was continued on broad-spectrum antibiotics and was referred to the orthopedic surgery and general surgery services for incision and drainage of the left hip joint and rectal abscesses respectively. Subsequent wound culture analysis returned positive for methicillin-sensitive Staphylococcus aureus (MSSA), for which the patient’s antibiotics were adjusted to nafcillin.

## Discussion

Septic arthritis is an acute and debilitating syndrome caused by inflammatory and infectious damage to a joint, particularly joints of the lower extremities [4]. This infectious etiology is usually a bacterial infection, with *Staphylococcus aureus* causing 42% of native joint septic arthritis [5]. The causes are usually hematologic seeding of the joint space or invasion of the joint space by the bacteria from adjacent tissues; joints are especially vulnerable to bacterial seeding due to easy passage of bacteria from the vasculature into joint tissue and synovial fluid [5]. Prosthetic implants also pose a risk for bacterial seeding and accumulation. Perirectal abscesses and fistulas usually originate from an infected anal canal [6]. Obstruction of glands at the base of the anal crypts at the level of the dentate line can lead to stasis, bacterial growth, and ultimately abscess formation. These abscesses can then extend through soft tissue and progress to a joint and ultimately cause septic arthritis and even osteomyelitis [6].

Patients suffering from septic arthritis typically present with fever, joint pain, limited range of motion secondary to pain, and in advanced cases, sepsis [2]. Often joints are warm and swollen, and patients are reluctant to move them due to severe pain [2]. While CT scanning is a modality that may help in ruling out abscesses, MRI offers superior sensitivity for abscesses, septic arthritis, and soft tissue changes. Treatment involves debridement of the affected joint and a three- to four-week course of antibiotics to treat the causative infection [2-3].

Nongonococcal septic arthritis caused by Staphylococci species results in approximately 50% of joint function preservation following antibiotics therapy. This is drastically improved with prompt drainage of infected joint fluid [2]. Mortality rates are up to 20%, but these rates are compounded by risk factors that can worsen prognosis, such as advanced age and compromised immunity [2]. Our case is unique in that our patient presented only with groin pain that was worse with walking, and was otherwise asymptomatic. Adjacent seeding of the joint is also a less common cause of acute septic arthritis, given that hematologic spread and inoculation of the joint due to joint injections are usually the causative events [7]. Additionally, given that the abscess was extending from the rectum to the hip, the bacteria found was MSSA but not *Escherichia coli*, which is commonly identified in perianal and perirectal areas [6].

## Conclusions

Septic arthritis is uncommon but can be fatal. Although the vast majority of cases result from hematogenous seeding of a joint, invasion of joint space from adjacent tissues is a cause that warrants diagnostic workup with imaging. Given that epidemiologic data support prompt drainage of the joint to preserve as much joint function as possible, recognition and accurate diagnosis become of paramount importance. In presenting this article, we hoped to encourage a higher degree of suspicion for infectious tract formation and resultant seeding of a joint in cases of septic arthritis.
